# Beneficial Effects on Abdominal Bloating with an Innovative Food-Grade Formulation of *Curcuma longa* and *Boswellia serrata* Extracts in Subjects with Irritable Bowel Syndrome and Small Bowel Dysbiosis

**DOI:** 10.3390/nu14030416

**Published:** 2022-01-18

**Authors:** Attilio Giacosa, Antonella Riva, Giovanna Petrangolini, Pietro Allegrini, Teresa Fazia, Luisa Bernardinelli, Gabriella Peroni, Mariangela Rondanelli

**Affiliations:** 1 CDI (Centro Diagnostico Italiano), 20147 Milan, Italy; attilio.giacosa@gmail.com; 2 R&D Department, Indena SpA, 20139 Milan, Italy; antonella.riva@indena.com (A.R.); giovanna.petrangolini@indena.com (G.P.); pietro.allegrini@indena.com (P.A.); 3 Department of Brain and Behavioral Science, University of Pavia, 27100 Pavia, Italy; teresa.fazia01@ateneopv.it (T.F.); luisa.bernardinelli@unipv.it (L.B.); 4 Endocrinology and Nutrition Unit, Azienda di Servizi alla Persona “Istituto Santa Margherita”, University of Pavia, 27100 Pavia, Italy; 5 IRCCS Mondino Foundation, 27100 Pavia, Italy; mariangela.rondanelli@unipv.it; 6 Department of Public Health, Experimental and Forensic Medicine, Unit of Human and Clinical Nutrition, University of Pavia, 27100 Pavia, Italy

**Keywords:** bloating, irritable bowel syndrome, dysbiosis, *Curcuma longa*, *Boswellia serrata*

## Abstract

Bloating is a symptom frequently reported by subjects with irritable bowel syndrome (IBS) and small bowel dysbiosis, and Low FODMAP’s diet (LFD) has been used to treat them. Extracts of *Curcuma* *longa* and *Boswellia* *serrata* share anti-inflammatory and antimicrobial effects that could be useful in the management of these clinical conditions. The aim of this study was to evaluate the efficacy of curcumin and boswellia extracts (as Curcumin Boswellia Phytosome, CBP) and LFD on the relief of abdominal bloating in IBS subjects with small bowel dysbiosis, in comparison to LFD alone, in a 30-day supplementation, randomized trial. IBS participants were randomized to either the intervention (500 mg bid of CBP and LFD) or control arm (LFD). Small bowel dysbiosis has been defined by an increase of urinary indican with normal urinary skatole. A total of 67 subjects were recruited. The intervention group (33 subjects) showed a significant decrease (*p* < 0.0001) of bloating, abdominal pain, and indican values at the end of the study, when compared to the control group (34 subjects). Moreover, the subjects of the intervention group showed a significantly better (*p* < 0.0001) global assessment of efficacy (GAE) as compared to controls. In conclusion, in subjects with IBS and small bowel dysbiosis, abdominal bloating can be successfully reduced with a supplementation with CBP and LFD.

## 1. Introduction

Bloating is one of the most frequent symptoms reported by patients with gastrointestinal functional disorders [1,2,3,4]. In particular, over 90% of subjects with irritable bowel syndrome (IBS) complain of bloating [5,6,7,8,9,10,11] and, in addition, 20–30% of the general population suffers this functional gastrointestinal discomfort [12,13,14]. Abdominal bloating is defined as trapped gas, abdominal pressure, fullness, and tightness, with frequent evidence of abdominal distension. Bloating has a negative effect on quality of life and favors the appearance of psychological distress [3].

Growing interest is currently dedicated to the potential correlation between abdominal bloating, irritable bowel syndrome, small bowel bacterial overgrowth (SIBO), and dysbiosis. IBS is characterized by gut microbial dysbiosis, and gut microbiota could play a key role in the pathogenesis of this syndrome [15]. Bloating has a multifactorial etiology and SIBO, which is an intestinal microbiome dysbiosis, appears to be commonly associated with this symptom and with IBS [16]. 

The antibiotic treatment of IBS is generally linked to Rome criteria amelioration in 48% of subjects [17]. Dysbiosis in IBS is characterized by a loss of microbial diversity and temporal instability mostly due to diet, antibiotic usage, bacterial overgrowth, immune activation, and low-grade inflammation. Growing evidence supports the efficacy of microbiota-directed therapies, such as diet, prebiotics, probiotics, and antibiotics, in the treatment of IBS [18]. In particular, it has been shown that a diet poor in Fermentable Oligosaccharides, Disaccharides, Monosaccharides, and Polyols (FODMAPs) is efficacious in the treatment of bloating in IBS patients [19,20,21]. 

With regard to botanicals, recent literature shows that curcumin and Boswellia extracts share interesting anti-inflammatory and gut antimicrobial effects [22,23,24,25,26,27]. Moreover, curcumin shows efficacy in the brain-gut axis regulation [28,29]. 

Based on these data, the aim of this study was to evaluate the efficacy of curcumin and boswellia extracts (as Curcumin Boswellia Phytosome, CBP) and low FODMAP’s diet (LFD) on the relief of abdominal bloating in IBS subjects with small bowel dysbiosis, in comparison to low FODMAP’s diet alone.

## 2. Materials and Methods

### 2.1. Study Design

This is a one-month (30 days) supplementation, randomized trial in which participants were randomized to either the supplement or control arm, performed at the Department of Public Health of the University of Pavia, Italy. The objective of the study was to evaluate the efficacy and safety of CBP in subjects with abdominal bloating, IBS, and small bowel dysbiosis. The study was conducted in accordance with the Declaration of Helsinki and the ICH Guidelines for Good Clinical Practice, following approval from the Local Independent Ethics Committee (Ethic code number: 0612/22052019). Written informed consent was obtained from each participant. The study was conducted from 5 February 2021, to 3 October 2021.

### 2.2. Population

Male and female subjects, with a diagnosis of IBS and small bowel dysbiosis who complained of bloating, were potential candidates for the study. The inclusion criteria were as follows: age 18–70 years; bloating reported as the main symptom; and a diagnosis of moderate IBS according to Rome III criteria, as abdominal pain for at least 1 day per week in the last 3 months that is associated with two or more of the following: related to defecation, associated with a change in stool form, or associated with a change in stool frequency [30,31]; or small bowel dysbiosis positive, defined by the increase of urinary indican values with normal skatole urinary concentration [32,33].

Exclusion criteria were: normal urinary indican values or increased urinary skatole values; subjects already on a LFD or other dietary restriction, such as gluten free diet or lactose free diet within the past 6 months; soy, nuts, or seafood allergies or insulin-dependent diabetes; known history of celiac disease, symptomatic diverticular disease, or inflammatory bowel disease or microscopic colitis; prior small bowel or colonic surgery or cholecystectomy; use of antibiotics, excluding topical, in the past 3 months; bloody diarrhea or severe vomiting; and a medical history of severe renal disease (defined as serum creatinine >1.5 mg/dL), or liver disease (defined as altered values of liver function tests).

### 2.3. Treatment and Concomitant Medications

Subjects included in the study received CBP 500 mg bid for 30 days. The choice of dose was based on previous clinical experience with CBP [34]. The possibility of combining curcumin and boswellia extracts in one single food-grade delivery system allows a treatment approach more suitable in terms of the number of tablets consumed.

Supplementation compliance was calculated as the ratio between the administered supplement (as determined by returned tablets) and the expected intake during the actual treatment period for each patient. All subjects (cases supplemented with CBP and controls) were instructed to follow nutritional recommendations, according to the low FODMAP’s diet by the same dietician [35]. The concomitant administration of any other drugs for the treatment of digestive disorders that could affect the results or interfere with the study supplementation was not allowed, including short-acting spasmolytics (e.g., butylscopolaminium bromide) and short-acting analgesics (e.g., paracetamol) or anxiolytics.

### 2.4. Clinical Evaluation

Study visits were scheduled at baseline (day 0) and at day 30. On both occasions all participants filled a questionnaire on the following:

(1) The score of abdominal bloating. On the questionnaire, participants were asked whether they felt “bloating/uncomfortably full”. There were four options that participants could choose: none (symptom did not occur), mild (symptom occurred but did not interfere with usual activities), moderate (occurrence of symptom somewhat interfered with usual activities), or severe (occurrence of symptom resulted in an inability to perform usual activities) [36].
(2) The score of the abdominal pain intensity. The score was defined using a validated visual analog scale score for pain (0 for ‘no pain’ to 10 for ‘most severe pain’) [37,38].

For all subjects the assessment of intestinal dysbiosis was performed by evaluating the urinary indican and skatole within 7 days prior to study inclusion and at the end of study. Urinary indican and skatole were considered normal at values lower than 10 mg/L and 10 µg/L, respectively [39].

At the end of the study, according to Kruis et al., a global assessment of efficacy (GAE) using a 4-point scale was fulfilled by each subject (1 for ‘ineffective’, 2 for ‘moderately effective’-slight improvement of complaints’, 3 for ‘effective’-marked improvement in symptoms’, and 4 for ‘very effective’-as good as no symptoms’) [40,41].

At each of the two visits, vital signs were checked and serum samples were collected. At the end of study urine samples were collected. Laboratory assessments were performed and analyzed with reference to the normal ranges. Participants were instructed to promptly report during the 30-day supplementation on any unwanted discomfort, intended as adverse event. Adverse events were likewise checked at the final visit.

### 2.5. Supplement Description

For the clinical study, the sunflower lecithin-based formulation of *Curcuma longa* L. and *Boswellia serrata* standardized extracts (CBP, 500 mg) was prepared by Indena S.p.A., Milan, Italy, as oblong-shaped, film-coated tablets, corresponding to a content of 17.0–23.0% *w*/*w* of curcumin and 7.0–11.0% *w*/*w* of boswellia extracts, respectively, by high performance liquid chromatography (HPLC) assay [34]. The active ingredient was a solid dispersion containing the standardized association of curcumin extract (≥90% as total curcuminoids assessed by HPLC assay) and boswellia extract (≥65.0% of total triterpenic acids assessed by HPLC assay). The remaining food-grade components of the phytosome tablets are calcium carbonate E170 (95DC M; Dr Paul Lohman^®^, Emmerthal, Germany), polyvinylpolypyrrolidone E1202 (Polyplasdone^™^ XL; Ashland, Austria, Germany, United States), sodium croscarmellose E468 (Solutab^®^ A-IP; Blanver Farmoquimica LTDA, Taboão da Serra, Brasil), silicon dioxide E551 (Syloid^®^ 244FP; Grace, Columbia), talc E553b (Mondo Minerals B.V., Amsterdam, Netherlands), magnesium stearate E470b (Ligafood^®^; Peter Greven, Bad Muenstereifel, Germany), and hydroxypropyl methylcellulose E463-based film-coating (Opadry^®^ Clear; Colorcon, United Kingdom). Before releasing, the film-coated tablets containing the food-grade lecithin formulation were tested for appearance, average mass, uniformity of mass, HPLC-content of curcumin and boswellia extracts, disintegration time, and microbiological quality.

### 2.6. Study Endpoints

The primary endpoint was the decrease in intensity of abdominal bloating. Secondary endpoints included (1) the change of urinary indican values, (2) the change in intensity of abdominal pain, (3) the number of subjects with complete bloating relief, and (4) the GAE as assessed by the subject at the end of the study. Safety endpoints included adverse events, laboratory results, and vital signs.

### 2.7. Statistical Analysis

The sample size was estimated for the primary endpoint bloating using Zhao’s formula for Wilcoxon Mann–Whitney test [42]. With an alpha of 0.05, a power of 0.90, an allocation rate 1:1, and assuming that after supplementation 40% of treated subjects had better score (i.e., 0 or 1) in abdominal bloating compared with 10% of control subjects, the calculated total sample size was 46. However, the number of subjects recruited was 67 in order to compensate for any withdrawal.

Differences between baseline variables in the two groups were investigated using *t*-tests for continuous variables and chi-squared test for categorical ones. 

First, the Wilcoxon Mann–Whitney test was used to assess if there were pre–post differences between the supplemented cases and control group in the bloating and on abdominal pain that are measured by a numerical rating scale from 0 to 3 and from 0 to 10, respectively. Secondly, a Cumulative Link Model (CLM), implemented in the ordinal R package, was performed in order to estimate the effect of the supplementation on bloating and on abdominal pain (ordinal variables) [43,44,45]. 

In the fitted model, the outcome Y represented the post-measurement, while the effect of the explanatory variables were 𝛽_1_ for the pre-measurement, 𝛽_2_ for the treatment group, 𝛽_3_ for age, and 𝛽_4_ for sex, according to the following equation:

𝑌 (Post Measurement) = 𝛽_1_ (pre-measurement) + 𝛽_2_ (Treatment Group) + 𝛽_3_ (Age) + 𝛽_4_ (Sex) + 𝑒

As for the secondary endpoint indican, measured on a continuous scale, after log-transforming and checking for normality both graphically and numerically, a linear model as in the above equation was fitted on the log-transformed variable. All the analysis was performed using R 3.5.1 statistical software [46]. Descriptive statistics are reported as Mean ± Standard Deviation (SD) or Standard Error (SE) and frequency distribution.

## 3. Results

**Figure 1:**
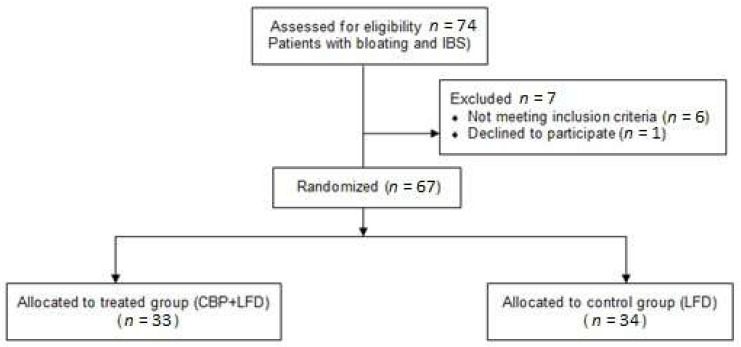
Flow diagram of the participants.

Seventy-four patients with bloating and IBS were screened, and 67 (90.5%), 51 females and 16 males, with mean (±SD) age of 43.09 (±15.10), were recruited and analyzed (Figure 1).

Six out of seven subjects were excluded because they did not meet the inclusion criteria: all of them had a urinary indican value within normal limits. One subject declined to participate.

Of the 67 enrolled subjects, 34 were randomly assigned to the control group, and 33 to the supplemented group. In Table 1, the descriptive statistics of the baseline variables for the two groups and the *p*-values for groups comparisons are reported. No statistically significant differences were observed between the two groups at baseline.

Results of the paired Wilcoxon Mann–Whitney test indicated a statistically significant pre–post difference between the two groups both for bloating (primary endpoint) (*p* < 0.0001) and for abdominal pain (secondary endpoint) (*p* < 0.0001). In Table 2, for each outcome and for each group the pre and post treatment mean and the estimate of the effect of the supplement, its standard error, and *p*-value for CLM or LM are reported. The estimate according to CLM should be interpreted as log odds of being in the higher level versus the combined middle and low categories of the outcome after supplementation in the treated group taking all the other variables in the model held constant. Both for bloating and abdominal pain, in the CBP group as compared to the control group a statistically significant effect of the proposed supplementation was observed, with a log odd respectively of 𝛽 = −6.11 (OR = 0.002 [95%CI 0.0001; 0.02], *p* < 0.0001) and 𝛽 = −5.23 (OR = 0.005 [95%CI 0.0003; 0.03], *p* < 0.0001) of being in the higher categories of score (corresponding to severe symptoms, at T1). 

As for indican, the result of the linear model on the log-transformed variable indicated a statistically significant effect of treatment in the CBP group (𝛽 = −0.52 and *p* < 0.0001), which corresponded to a decreasing of indican of 40% as compared to control group. 

In Table 3, the frequency of GAE measured in T1 is reported; a statistically significant difference (*p* < 0.0001) was observed between the two groups.

## 4. Discussion

This study clearly shows that the supplementation of CBP and LFD is associated with a significant decrease of bloating in subjects with IBS and small bowel dysbiosis as compared to controls treated with LFD alone. This finding demonstrates the crucial and positive role of CBP in achieving this primary outcome. At the end of the study, 33 subjects of the intervention group reported no or a mild degree of bloating, in comparison to only 10 subjects in the control group. According to our knowledge, previous clinical data on this effect of extracts of *Curcuma longa* and *Boswellia serrata* are not available and these results indicate a new potential option for the relief of bloating in IBS and small bowel dysbiosis subjects.

LFD has been utilized to ameliorate bloating discomforts, as well as IBS and SIBO in various studies, with promising results [19,20,21]. In our study LFD did not appear to be efficacious for bloating relief in IBS subjects with dysbiosis, while the concomitant supplementation with CBP for 30 days was followed by a significant reduction of bloating. 

A retrospective study published by Gibson et al. [47] and other controlled prospective studies [21,48,49], showed the efficacy of LFD in reducing bloating and other abdominal symptoms such as abdominal pain, flatulence, and diarrhea in Australian and New Zealand IBS patients. Furthermore, positive effects on bloating and IBS after following LFD have been reported with other studies in North American, British, and Nordic countries [50]. The marked difference between the Mediterranean and western diet, typically followed in most of the countries where the LFD had previously been recommended, could partly explain the different results. In Italy, in particular, where our study was conducted, the usual diet is much richer in gluten and less rich in lactose, compared to a typical western diet.

A similarity with our outcomes comes from the study of Pedersen et al. [50]. In that trial the group of IBS patients treated with standard management such as laxatives, antispasmodic, antidiarrheal and antidepressant drugs plus LFD showed a significantly higher efficacy when compared with LFD alone [50]. The improved response to combined different approaches could be explained by the complexity of IBS pathogenic pathway and symptom etiology, with requirement of several medications that are effective against specific pathogenic mechanisms. 

In our trial positive effects of the addition of CBP to LFD have been obtained when abdominal pain was considered. As a matter of fact, abdominal pain decreased significantly only in that group. LFD alone was not efficacious to reduce abdominal pain in IBS subjects with small bowel dysbiosis, thus, underlining the positive and fundamental role of CBP. 

Takamura and Pimentel showed that, as a consequence of intestinal dysbiosis, subjects with IBS symptoms, including bloating, may have increased intestinal permeability, dysmotility, chronic inflammation, autoimmunity, decreased absorption of bile salts, and even altered enteral and central neuronal activity [16]. Thus, from the pathogenic point of view, we see relevant interest in the reduction of small bowel dysbiosis that has been demonstrated in our study, taking into account the significant decrease of urinary indican, after supplementation with CBP and LFD. The potential explanation of this observation is due to the anti-inflammatory and, mostly, the antimicrobial activities of both the curcumin and boswellia extracts [22,23,24,25,26]. The demonstrated antimicrobial effects of curcuma and boswellia extracts could target the bacterial imbalance involved in the small bowel dysbiosis associated to bloating. 

Antibiotics are widely used to treat bloating in order to counteract intestinal dysbiosis that could be responsible for increasing gas production. In various trials rifaximin has been reported to be significantly better than placebo in reducing bloating in patients with IBS, thus, confirming the efficacy of anti-bacterial therapy [51,52,53]. A “natural antibiotic” such as CBP could enlarge the available tools for IBS and SIBO management, taking into consideration also its tolerability as shown in clinical use. Bloating is the most common symptom associated with disorders of brain–gut interaction (i.e., functional bowel disorders), such as irritable bowel syndrome, a gastrointestinal disorder characterized by abdominal pain and altered bowel habits which affects up to 11% of world population [5]. Curcumin extracts have been demonstrated to act efficaciously on the brain–gut axis and this could be an additional mechanistic effect that could explain the positive results obtained with CBP in our study [28,29].

Up to now there has been no evidence-based algorithm for treating subjects with bloating. The treatment options are diet, exercise, pharmacological medications, probiotics, antibiotics, smooth muscle antispasmodics, osmotic laxatives, prokinetic agents, chloride channel activators, and tricyclic antidepressants. This new, natural approach based on supplementation with extracts of *Curcuma longa* and *Boswellia serrata* appears to be useful for abdominal bloating in subjects with IBS and small bowel dysbiosis following a LFD. 

The positive outcome of this botanical management is confirmed by the participants’ global assessment of efficacy (GAE) that showed a significant difference when cases and controls were compared. At the end of the study, 93.9% of the CBP group were classified in grade 3 (‘effective-marked improvement in symptoms’) or in grade 4 (‘very effective-as good as no symptoms’) of GAE, while this result was obtained only by 14.7% of controls. The phytosome technology is a 100% food-grade technology applied to natural products in order to obtain a solid dispersion by mixing a carrier like phospholipids (i.e., soy or sunflower lecithin) and the active principles, in order to increase the exposure area of natural molecules or extracts to the gastrointestinal layer. Recently, Del Rio et al. demonstrated that phytosome formulation notably influenced the biotransformation of curcuminoids since the fermentation of lecithin-curcuminoids by fecal human microbiota led to more efficient production of curcuminoid catabolites [54].

Some limitations of our study need to be taken into account. The subjects have been randomized to the two different treatment choices, but there was lack of blinding. Furthermore, the control group did not receive placebo but LFD and this constitutes a weakness of the study, when referring to the large placebo responses in IBS revealed in other studies. A final limitation of the study is that the adherence with LFD has not been checked in both the intervention and control groups.

## 5. Conclusions

In conclusion, the strength of our study is that, to the best of our knowledge, this is the first controlled clinical research project in which abdominal bloating in IBS subjects with small bowel dysbiosis has been successfully treated with a concomitant supplementation with extracts of *Curcuma longa* and *Boswellia serrata* (as curcumin boswellia phytosome) and LFD. Moreover, this is one of the few studies in which abdominal bloating has been evaluated as a primary endpoint in IBS patients and one of the few studies in which LFD has been tested to treat IBS in a Mediterranean country.

Our results should be confirmed by larger trials, with longer duration of supplementation.

## Figures and Tables

**Table 1:** Baseline participant characteristics.

	Treated Group (*n* = 33) Mean (SD)	Control Group (*n* = 34) Mean (SD)	*p*-Value ^a^
Age (years)	42.82 (14.58)	43.36 (15.84)	0.88
Sex			
Female	26 (76.47%)	25 (75.76)	1
Male	8 (23.53%)	8 (24.24)	

^a^*p*-value for between group comparisons.

**Table 2:** Effect of the supplementation on the primary and secondary outcomes.

	Group	Mean (SD) T0	Mean (SD) T1	Estimate	SE	*p*-Value *
Primary endpoint						
Bloating	Treated Control	2.94 (0.24) 2.76 (0.43)	0.15 (0.36) 1.85 (0.74)	−6.11	1.18	<0.0001
Secondary endpoints						
Abdominal Pain	Treated Control	7.39 (2.97) 7.47 (2.38)	1.00 (1.44) 4.71 (1.91)	−5.23	1.15	<0.0001
Indican (mg/L)	Treated Control	92.39 (53.52) ^a^ 90.14 (54.82)	52.38 (27.22) ^a^ 85.53 (54.24)	−0.52	0.11	<0.0001

* All models are adjusted for sex and age. Bloating and Abdominal Pain results were obtained fitting CLM, while Indican results were obtained fitting LM. ^a^ Statistics obtained in the original scale.

**Table 3:** Frequencies of GAE (global assessment of efficacy) at the end of study.

Variable	*N*(%) All Sample	*N*(%) Control	*N*(%) Treated	*p*-Value
GAE				
1	17 (25.37%)	17 (50%)	0 (0%)	<0.0001
2	14 (20.89%)	12 (35.29%)	2 (6.1%)	
3	17 (25.37%)	5 (14.71%)	12 (36.36%)	
4	19 (28.36%)	0 (0%)	19 (57.58%)	

## Data Availability

The data presented in this study are available in the text file.
